# Timing of delivery in women with diabetes: A population‐based study

**DOI:** 10.1111/aogs.13761

**Published:** 2019-12-08

**Authors:** Amy Metcalfe, Jennifer A. Hutcheon, Yasser Sabr, Janet Lyons, Jason Burrows, Lois E. Donovan, K. S. Joseph

**Affiliations:** ^1^ Department of Obstetrics and Gynecology University of Calgary Calgary Alberta Canada; ^2^ Department of Medicine University of Calgary Calgary Alberta Canada; ^3^ Department of Community Health Sciences University of Calgary Calgary Alberta Canada; ^4^ Department of Obstetrics and Gynecology University of British Columbia Vancouver British Columbia Canada; ^5^ School of Population and Public Health University of British Columbia Vancouver British Columbia Canada; ^6^ Department of Obstetrics and Gynecology King Saud University Riyadh Saudi Arabia

**Keywords:** diabetes mellitus, gestational diabetes, neonatal outcome, obstetric delivery, pregnancy

## Abstract

**Introduction:**

Women with diabetes, and their infants, have an increased risk of adverse events due to excess fetal growth. Earlier delivery, when fetuses are smaller, may reduce these risks. This study aimed to evaluate the week‐specific risks of maternal and neonatal morbidity/mortality to assist with obstetrical decision making.

**Material and methods:**

In this population‐based cohort study, women with type 1 diabetes (n = 5889), type 2 diabetes (n = 9422) and gestational diabetes (n = 138 917) and a comparison group without diabetes (n = 2 553 243) who delivered a singleton infant at ≥36 completed weeks of gestation between 2004 and 2014 were identified from the Canadian Institute of Health Information Discharge Abstract Database. Multivariate logistic regression was used to determine the week‐specific rates of severe maternal and neonatal morbidity/mortality among women delivered iatrogenically vs those undergoing expectant management.

**Results:**

For all women, the absolute risk of severe maternal morbidity/mortality was low, typically impacting less than 1% of women, and there was no significant difference in gestational age‐specific severe maternal morbidity/mortality between iatrogenic delivery and expectant management among women with any form of diabetes. Among women with gestational diabetes, iatrogenic delivery was associated with an increased risk of neonatal morbidity/mortality compared with expectant management at 36 and 37 weeks’ gestation (76.7 and 27.8 excess cases per 1000 deliveries, respectively) and a lower risk of neonatal morbidity/mortality at 38, 39 and 40 weeks’ gestation (7.9, 27.3 and 15.9 fewer cases per 1000 deliveries, respectively). Increased risks of severe neonatal morbidity following iatrogenic delivery compared with expectant management were also observed for women with type 1 diabetes at 36 (98.3 excess cases per 1000 deliveries) and 37 weeks’ gestation (44.5 excess cases per 1000 deliveries) and for women with type 2 diabetes at 36 weeks’ gestation (77.9 excess cases per 1000 deliveries) weeks.

**Conclusions:**

The clinical decision regarding timing of delivery is complex and contingent on maternal‐fetal wellbeing, including adequate glycemic control. This study suggests that delivery at 38, 39 or 40 weeks’ gestation may optimize neonatal outcomes among women with diabetes.

**Abbreviations**
CCI: Canadian Classification of Health Interventions
DCS, Diabetes Complications Severity Index
GDM: gestational diabetes
ICD‐10‐CA: International Classification of Disease Version 10 Canadian Modification

**Key message**

Iatrogenic delivery is associated with increased risk of neonatal morbidity/mortality for women with diabetes in the late preterm/early term period. However, iatrogenic delivery at 38, 39 or 40 weeks’ gestation was associated with lower rates of neonatal morbidity/mortality for women with gestational diabetes.

## 1 INTRODUCTION

Diabetes in pregnancy has been shown to increase the risk of perinatal mortality, morbidity and congenital anomalies.^1^, ^2^ Risks of adverse outcomes are not constant across all types of diabetes; women with type 1 diabetes typically have the highest rate of adverse outcomes, followed by women with type 2 diabetes and women with gestational diabetes (GDM).^3^ Fetuses exposed to diabetes in utero appear to have a different growth trajectory, with excess weight in the trunk and shoulders, compared with fetuses of women without diabetes;^4^, ^5^ this places them at an increased risk for macrosomia and birth injuries such as shoulder dystocia and brachial plexus injuries, even after controlling for fetal size.^6^, ^7^ These risks can be reduced with good glycemic control pre‐conceptionally and throughout pregnancy.^1^, ^8^ The Hyperglycemia and Adverse Pregnancy Outcomes (HAPO) study shows that there is a continuous linear association between maternal glucose levels and birthweight.^9^

It has been hypothesized that iatrogenic delivery at earlier term gestation reduces the risk of neonatal complications (as opposed to expectant management).^6^ The clinical decision about when to deliver women with diabetes depends on many maternal and fetal factors and the potential risk of adverse outcomes.^6^, ^10^, ^11^ A 2018 Cochrane systematic review attempted to determine the optimal timing of delivery for women with preexisting diabetes but concluded that there was insufficient evidence to answer this question as no trials had been published in this population.^12^ A similar Cochrane systematic review, focusing on women with GDM, also concluded that there was limited data to support clinical decision making.^13^ To date, only three small randomized controlled trials of 200 women,^14^ 425 women,^15^ and 100 women,^16^ respectively, have been published on timing of delivery among women with diabetes. Among women with insulin‐requiring diabetes in pregnancy, Kjos et al^14^ found that infants born following expectant management were significantly larger than infants born following induction of labor. The GINEXMAL trial found no significant differences in cesarean delivery rates or severe maternal/neonatal morbidity between women with labor GDM randomized to induction of labor or expectant management between 38^+0^ and 39^+0^ weeks of gestation.^15^ However, infants born following induction of labor were significantly more likely to have hyperbilirubinemia.^15^ Finally, the third small trial by Worda et al^16^ also did not observe significant differences in large‐for‐gestational age or cesarean delivery following induction of labor at 38 vs 40 weeks of gestation among women with insulin‐controlled GDM. 

To our knowledge, no study has examined the impact of gestational age at delivery on maternal and neonatal morbidity in women with diabetes, and how this varies by type of diabetes. As such, this study aimed to quantify the week‐specific risks of maternal and neonatal morbidity and mortality in women who delivered iatrogenically following induction of labor or pre‐labor cesarean section compared with expectant management for women with type 1 diabetes, type 2 diabetes and GDM.

## 2 MATERIAL AND METHODS

Our study population was drawn from all non‐anomalous singleton hospital births in Canada (except Quebec) at or beyond 36 weeks’ gestation from 2004 to 2014. Individual level de‐identified delivery data were obtained from the Canadian Institute of Health Information Discharge Abstract Database. Data were entered into the Discharge Abstract Database by trained health coders and this database has been validated for use in perinatal epidemiologic studies.^17^ Delivery was identified using International Classification of Disease Version 10 Canadian Modification (ICD‐10‐CA) codes for live births and stillbirths. Mother‐infant dyads were excluded if delivery occurred at <36 weeks’ or >42 weeks’ gestation (as delivery prior to this stage would not be clinically recommended in the absence of other issues impacting maternal or fetal health), gestational age data were missing or the infant had a congenital anomaly.

The following hierarchical algorithm was used to classify women with diabetes: codes for Type 1 diabetes (Mother: ICD‐10‐CA E10.x, O24.5) superseded all other diabetes codes, and codes for Type 2 diabetes (Mother: ICD‐10‐CA E11.x, O24.6) superseded GDM codes. Women with codes for GDM (Mother: ICD‐10‐CA O24.8; Infant: ICD‐10‐CA P70.0) who did not have other codes for preexisting diabetes were classified as having GDM. The comparison group consisted of women with none of the above‐mentioned codes for diabetes. Women with impaired glucose tolerance but who had not been diagnosed with type 1, type 2 or GDM, were therefore included in the comparison group. For each woman with type 1 or type 2 diabetes, a Diabetes Complications Severity Index (DCSI) score was calculated. The DCSI is an ICD‐10 based scoring system that assigns zero (no complications), one (no severe complications, but at least one mild complication) or two (one or more severe complications) points based on the presence of any of seven diabetic complications, namely, cardiovascular, cerebrovascular or metabolic complications or nephropathy, neuropathy, peripheral vascular disease or retinopathy.^18^

**Figure 1:**
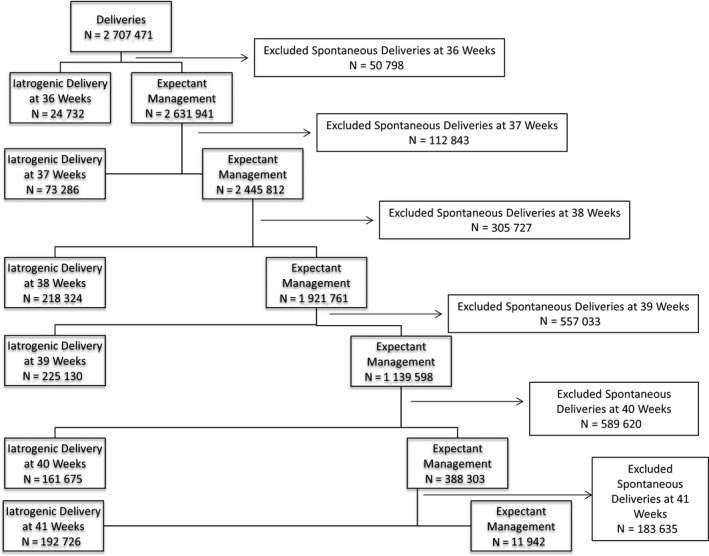
Study flow diagram

Women were categorized as undergoing iatrogenic delivery if codes were present for labor induction or pre‐labor cesarean delivery. Labor induction was identified based on a Canadian Classification of Health Interventions (CCI) code. Currently, no CCI or ICD‐10‐CA codes exist to identify pre‐labor cesarean delivery;^19^ however, a validated algorithm has been developed and used in multiple studies to identify labor using ICD‐9‐CM codes.^20^, ^21^, ^22^ Women were classified as having a pre‐labor cesarean section if they had a CCI code for cesarean delivery and no ICD‐10‐CA codes were present indicating that labor occurred. Women who did not deliver in a specific week of gestation were categorized as undergoing expectant management at that week of gestation and served as the comparison group, regardless of whether they later delivered following spontaneous onset of labor or iatrogenic delivery. Women who experienced spontaneous labor onset and subsequent delivery at a given week of gestation were excluded from the comparison (expectant management) group for that particular week of gestation (Figure 1; Figure S1).

The primary outcomes were a composite of maternal mortality or severe maternal morbidity, and severe neonatal morbidity or mortality. Maternal death or severe maternal morbidity was defined as the occurrence of one or more of the following conditions/procedures in the immediate perinatal period: maternal death prior to discharge, obstetric embolism, obstetric shock, postpartum hemorrhage with hysterectomy or other procedures to control bleeding, sepsis, thromboembolism or uterine rupture. The specific ICD‐10‐CA and CCI codes used can be found in Table S2. The above‐listed outcomes were selected to reduce the probability of confounding by indication, as they are unlikely to be an indication for labor induction or pre‐labor cesarean delivery but may be an unintended consequence of these procedures.^19^ Severe neonatal morbidity or mortality was defined as: birth asphyxia, fetal asphyxia, intraventricular hemorrhage (grade 3 or 4), neonatal convulsions, other disturbances of cerebral status of newborn, respiratory distress syndrome, birth injury to central nervous system, birth injury to peripheral nervous system, birth injury to skeleton, fetal fracture of humerus or clavicle to facilitate delivery, shoulder dystocia, stillbirth or neonatal death. The specific ICD‐10‐CA and CCI codes used can be found in Table S2.

### 2.1 Statistical analyses

At each gestational age, the observed rate of each outcome was calculated per 100 deliveries following iatrogenic delivery and per 100 ongoing pregnancies following expectant management. These separate denominators were chosen as they mimic the real‐life decisions that clinicians make (ie, iatrogenic delivery or expectant management). Of note, a woman who was expectantly managed at 37 weeks of gestation could later be delivered iatrogenically at 38 weeks if her clinical situation changed. These observed rates were used to generate unadjusted risk differences and risk ratios. Logistic regression models were derived to calculate adjusted risk ratios, risk differences, and absolute predicted risks for each outcome at each week of gestation (ie, at each week of gestation, the risk of adverse outcomes following iatrogenic delivery was compared with the same risk among ongoing pregnancies). All models were adjusted for year, parity and the obstetric comorbidity score (a validated composite risk score that included preexisting chronic disease, pregnancy‐associated disease and maternal age^23^, ^24^). Although preexisting diabetes is a component of the obstetric comorbidity score, it was not included in the index for this study, as the type of diabetes was the primary exposure variable. Models examining neonatal outcomes were further adjusted for infant sex. As there is an increased risk of recurrence for many adverse obstetric events, a sensitivity analysis was conducted, restricted to the first birth during the study period for all women. All analyses was conducted using STATA SE Version 14 (StataCorp.).

### 2.2 Ethical approval

Ethics approval for this study was obtained from the Conjoint Health Research Ethics Board at the University of Calgary on 3 September 2014 (REB14‐1314). Patients were not directly involved in this study.

## 3 RESULTS

**Figure 2:**
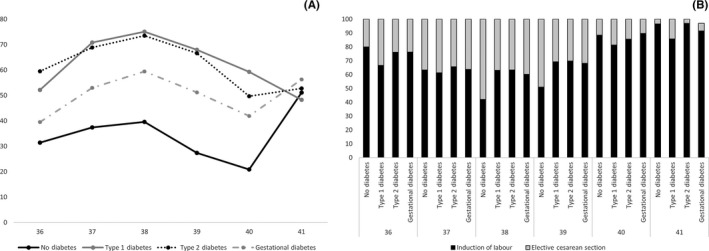
Iatrogenic delivery by week of gestation. (A) Incidence of iatrogenic delivery by gestational age and type of diabetes. (B) Method of iatrogenic delivery by gestational age and type of diabetes

Overall, 3 029 523 women delivered during the study period and their records could be linked to their infants. Women were excluded due to missing information on gestational age (n = 3857), delivery at <35 weeks of gestation (n=142 398) or >42 weeks of gestation (n= 353), having an infant with a congenital anomaly (n=141 883). Overall, 2 707 471 women were included in this study; including 5889 women with type 1 diabetes, 9422 women with type 2 diabetes and 138 917 women with GDM. The prevalence of diabetes‐related complications was low—97.0% of women with type 1 diabetes and 99.1% of women with type 2 diabetes had a DCSI of zero, indicating no diabetes‐related complications. Women with any form of diabetes were approximately twice as likely to have an iatrogenic delivery compared with women without diabetes and were also more likely to experience an adverse maternal or neonatal outcome (Table 1). Similar characteristics were observed when the analysis was restricted to first births during the study period (Table S3). The incidence of iatrogenic delivery by gestational age differed by type of diabetes, and elective cesarean section was used more frequently in women with any type of diabetes than in women without diabetes (Figure 2).

**Table 1:** Characteristics of women delivering singleton non‐anomalous infants ≥36 wk of gestation in Canadian hospitals (excluding Quebec) between 2004 and 2014

Characteristic	Women without diabetes n = 2 553 243	Women with type 1 diabetes n = 5889	Women with type 2 diabetes n = 9422	Women with gestational diabetes n = 138 917
Maternal age (y), mean (SD)	29.3 (5.6)	29.8 (5.3) *P *<* *0.001	32.6 (5.5) *P *<* *0.001	32.1 (5.3) *P *<* *0.001
Gestational age at delivery (wk), mean (SD)	39.2 (1.2)	37.6 (1.0) *P *<* *0.001	37.9 (1.1) *P *<* *0.001	38.6 (1.2) *P *<* *0.001
Iatrogenic delivery*, % (95% CI)	32.1 (32.1‐32.2)	68.7 (67.5‐69.9) *P *<* *0.001	67.9 (66.9‐68.8) *P *<* *0.001	52.2 (51.9‐52.5) *P* < 0.001
Mode of delivery, % (95% CI)				
Spontaneous vaginal	63.7 (63.7‐63.8)	30.7 (29.5‐31.9)	42.3 (41.3‐43.3)	53.0 (52.7‐53.2)
Operative vaginal	10.6 (10.6‐10.7)	9.9 (9.2‐10.7)	7.3 (6.8‐7.9)	10.0 (9.8‐10.1)
Cesarean section	25.6 (25.6‐25.7)	59.4 (58.1‐60.6) *P* < 0.001	50.4 (49.4‐51.4) *P* < 0.001	37.0 (36.8‐37.3) *P* < 0.001
Hypertensive disorders of pregnancy, % (95% CI)	4.8 (4.8‐4.8)	17.7 (16.7‐18.8) *P* < 0.001	15.3 (14.6‐16.1) *P* < 0.001	9.3 (9.1‐9.4) *P* < 0.001
Parity, % (95% CI)				
Nulliparous	42.0 (41.9‐42.1)	44.5 (43.0‐45.9)	28.8 (27.8‐29.8)	35.2 (34.9‐35.5)
Multiparous, no history of prior cesarean section	45.4 (26.3‐28.9)	27.6 (26.3‐28.9)	45.4 (44.3‐46.5)	43.9 (43.6‐44.3)
Multiparous, prior cesarean section	12.6 (12.6‐12.6)	28.0 (26.7‐29.3) *P* < 0.001	25.8 (24.8‐26.8) *P* < 0.001	20.9 (20.6‐21.1) *P* < 0.001
Diabetes complications severity index score, mean (SD)	—	3.5 (.2)	1.4 (.2)	—
Male infant sex, % (95% CI)	50.6 (50.6‐50.7)	49.9 (48.6‐51.2) *P* < 0.25	50.2 (49.2‐51.3) *P* < 0.33	51.5 (51.2‐51.7) *P* < 0.001
Infant birthweight (g), mean (SD)	3450.3 (481.1)	3710 (615.8) *P* < 0.001	3562.7 (623.4) *P* < 0.001	3446.4 (526.6) *P* < 0.04
Large for gestational age infant >97th percentile, %, 95% CI	9.2 (9.2‐9.3)	44.6 (43.3‐48.9) *P* < 0.001	31.3 (30.3‐32.2) *P* < 0.001	15.5 (15.3‐15.7) *P* < 0.001
Severe maternal morbidity/mortality, % (95% CI)	0.4 (0.4‐0.4)	0.6 (0.4‐0.8) *P* < 0.02	0.8 (0.6‐1.0) *P* < 0.001	0.5 (0.5‐0.6) *P* < 0.001
Severe neonatal morbidity/mortality, % (95% CI)	7.7 (7.7‐7.8)	20.8 (19.7‐21.8) *P* < 0.001	15.6 (14.9‐16.4) *P* < 0.001	9.8 (9.7‐10.0) *P* < 0.001

Chi‐square tests were used to assess differences between groups for categorical variables, and ANOVA and *t *tests were used to assess differences between groups for continuous variables. *P* values compare women with diabetes with the general population.

* Defined as a delivery following induction of labor or pre‐labor cesarean delivery.

**Figure 3:**
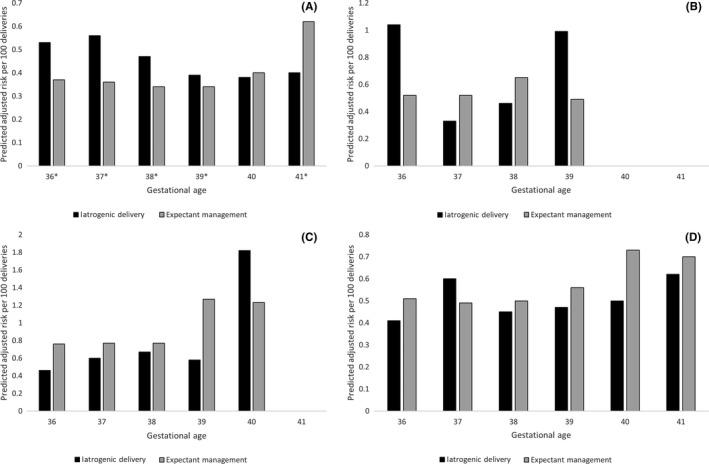
Predicted adjusted risk of maternal morbidity/mortality per 100 deliveries for (A) women without diabetes, (B) women with type 1 diabetes, (C) women with type 2 diabetes and (D) women with gestational diabetes. Estimates are adjusted for year, obstetric comorbidity score and parity. *Indicates statistically significant results. Maternal death or severe maternal morbidity was defined as the occurrence of one or more of the following conditions/procedures in the immediate postpartum period: maternal death prior to discharge, obstetric embolism, obstetric shock, postpartum hemorrhage with hysterectomy or other procedures to control bleeding, sepsis, uterine rupture, thromboembolism

For all women, the absolute risk of severe maternal morbidity/mortality was very low, typically impacting less than 1% of women regardless of whether they were iatrogenically delivered or expectantly managed (Figure 3; Table S4). The absolute risk of severe maternal morbidity/mortality generally displayed a U‐shaped relation with gestational age. Among women without diabetes, iatrogenic delivery was associated with a significantly higher adjusted risk of severe maternal morbidity/mortality at 36, 37, 38, and 39 weeks of gestation compared with expectant management, and a decreased risk of severe maternal morbidity/mortality at 41 weeks of gestation. For example, at 36 weeks, there were 1.6 excess cases of severe maternal morbidity/mortality per 1000 deliveries following iatrogenic delivery compared with expectant management, whereas at 39 weeks, there were .5 excess cases of maternal morbidity/mortality per 1000 deliveries. No significant differences were observed between iatrogenic delivery and expectant management in gestational age‐specific rates of severe maternal morbidity/mortality among women with any form of diabetes. Results were similar when restricted to first births during the study period (Table S5).

**Figure 4:**
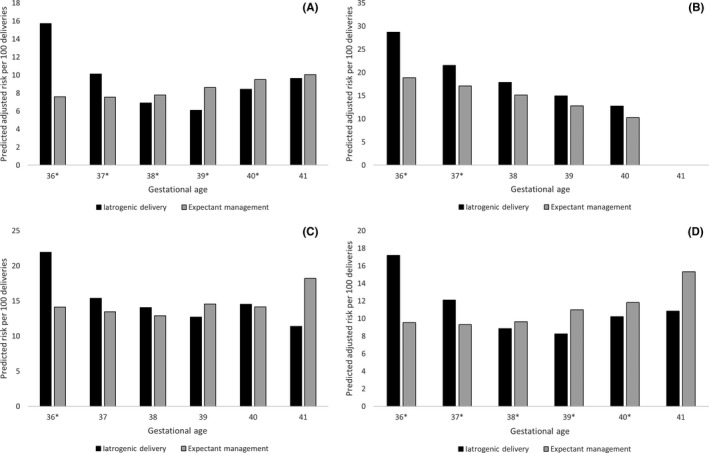
Predicted adjusted risk of neonatal morbidity/mortality per 100 deliveries for infants born to (A) women without diabetes, (B) women with type 1 diabetes, (C) women with type 2 diabetes and (D) women with gestational diabetes. Estimates are adjusted for year, obstetric comorbidity score, parity and infant sex. ^*^Indicates statistically significant results. Severe neonatal morbidity or mortality was defined as the occurrence of one or more of the following conditions/procedures during the birth hospitalization: birth asphyxia, fetal asphyxia, intraventricular hemorrhage (grade 3 or 4), neonatal convulsions, other disturbances of cerebral status of newborn, respiratory distress syndrome, birth injury to central nervous system, birth injury to peripheral nervous system, birth injury to skeleton, fetal fracture of humerus or clavicle to facilitate delivery, shoulder dystocia, stillbirth, neonatal death

The absolute risk of severe neonatal morbidity/mortality was much higher than the risk of maternal morbidity/mortality and also displayed a U‐shaped relation with gestational age (Figure 4; Table S6). Among women without diabetes and women with GDM, iatrogenic delivery was associated with an increased adjusted risk of neonatal morbidity/mortality at 36 and 37 weeks of gestation compared with expectant management. However, such women had a lower adjusted risk of neonatal morbidity/mortality at 38, 39 and 40 weeks of gestation following iatrogenic delivery. For example, at 36 weeks, there were 81.3 and 76.7 excess cases of neonatal morbidity/mortality per 1000 deliveries in women without diabetes and women with GDM, respectively, following iatrogenic delivery compared with expectant management, whereas at 39 weeks, there were 25.1 and 27.3 fewer cases of neonatal morbidity/mortality per 1000 deliveries in women without diabetes and with GDM, respectively, following iatrogenic delivery compared with expectant management. Increased risks of severe neonatal morbidity/mortality following iatrogenic delivery were also observed for women with type 1 diabetes at 36 (98.3 excess cases per 1000 deliveries) and 37 weeks of gestation (44.5 excess cases per 1000 deliveries) and for women with type 2 diabetes at 36 weeks of gestation (77.9 excess cases per 1000 deliveries) compared with expectant management. Results were similar when restricted to first births during the study period (Table S7).

## 4 DISCUSSION

Our study suggests that there may be some benefit to the infant if delivery of women with diabetes occurred at 38, 39 or 40 weeks’ gestation. Timing of delivery for a woman with diabetes is a complex issue for patients and care providers and depends on several individual and contextual factors. The results of this study may be useful in supporting discussions on the risks and benefits of early delivery. However, one must recognize that even with the use of modern diabetes management tools, ideal glycemic control in women with type 1 diabetes is elusive, neonatal complications rates remain high^25^ and stillbirth is associated with poor glycemic control in women with type 1 or type 2 diabetes.^11^

Currently no consensus exists in the literature or in clinical guidelines as to the optimal timing of delivery for women with diabetes.^2^, ^6^ Clinical practice patterns vary across provider types and settings. A survey of clinicians participating in an international conference on the management of patients with GDM found that for patients with diet‐controlled GDM, 10% delivered their patients at 38 weeks of gestation, 14% at 39 weeks, 57% at 40 weeks and 16% at 41 weeks.^26^ For patients with medication‐controlled GDM, 28% of respondents delivered their patients at 38 weeks of gestation, 46% at 39 weeks and 19% at 40 weeks.^26^

The bulk of the literature on the timing of delivery in women with diabetes comes from retrospective observational studies and suffers from methodological limitations such as a failure to adjust for important confounders, an inability to distinguish diabetes sub‐types and a lack of information on glycemic control.^27^ A study using California vital statistics data from 1997 to 2006 found that the risk of stillbirth increased for all women with increasing gestational age, and that women with GDM had an increased risk of stillbirth compared with women without GDM at 36‐41 weeks of gestation.^28^ Even though the relative risk of stillbirth is increased for women with GDM, the absolute risk of stillbirth remains low—this translates to large numbers of women needing to be delivered at earlier gestational ages to prevent a single case of stillbirth (4435 at 38 weeks of gestation, 1518 at 39 weeks, and 1311 at 40 weeks).^28^ Results from the present study examining morbidity patterns corroborate these findings, and show a decreased risk of neonatal morbidity for women with GDM who delivered at 38, 39 or 40 weeks of gestation. The present study also shows the impact that late preterm and early term birth has on child health: infants born following iatrogenic delivery at 36 and 37 weeks of gestation for women with GDM and type 1 diabetes had an increased risk of severe neonatal morbidity/mortality.

Our study, and the bulk of the literature in this area, supports delivery at 38, 39 or 40 weeks of gestation for women with GDM. However, there is insufficient evidence on timing of delivery for women with type 1 and type 2 diabetes. Our findings suggest no maternal benefit and little or no additional neonatal benefit of iatrogenic delivery at 39 rather than 38 weeks of gestation for women with type 1 or type 2 diabetes. However, these women have a much greater risk of stillbirth compared with women with GDM, and ideal glycemic control is challenging to achieve in women with type 1 diabetes. For this reason, there is little justification for delaying delivery of women with preexisting diabetes beyond 38 weeks of gestation, particularly in the presence of poor glycemic control, which is an independent risk factor for stillbirth.^11^

This population‐based observational cohort study suffers from limitations common to this design. While a randomized controlled trial is the ideal study design to investigate this question, given the recruitment difficulties faced by the investigators of the GINEXMAL trial of delivery timing for women with GDM at term,^15^ it is unlikely that another randomized controlled trial will be conducted on this issue. Our study lacked data on important confounders, namely glycemic control, estimated fetal weight and obstetric history, which are critical to consider when determining the optimal timing of delivery for a given patient. We are unable to rule out the possibility of confounding by indication by conclusively establishing that each of the elements that constituted our definitions of severe maternal and neonatal morbidity and mortality were not an indication for delivery. We also lack data on which diagnostic criteria were used to diagnosis GDM; these have changed over time and impacted the proportion of women diagnosed with GDM.^29^ However, hospital delivery records accurately identify women with preexisting and GDM (the primary exposure in this study). A systematic review found that GDM (sensitivity 71.0‐81.3%, specificity 99.4‐99.6%, positive predictive value 50.0‐88.8%) and preexisting diabetes (sensitivity 78.0‐95.3%, specificity 99.4‐100.0%, positive predictive value 94.0‐97.6%) were accurately coded in birth certificate and hospital discharge data in the USA.^30^ A Canadian study found that the Discharge Abstract Database had a sensitivity of 88.5% and a specificity of 99.9% in identifying preexisting diabetes in pregnancy.^31^ As we only had access to hospitalization data, the prevalence of diabetes‐related complications is likely underestimated.^24^ Due to sample size issues, we were also not able to examine perinatal mortality or maternal mortality in isolation. Another study examined the risk of perinatal mortality by gestational age among women with GDM and found that mortality risks were lower following iatrogenic delivery after 39 weeks of gestation; however, the number needed to treat was high due to the small absolute risk of mortality.^28^

The above‐mentioned limitations notwithstanding, this study is the first to compare the risks of iatrogenic delivery and expectant management by diabetes type. This is important, as previous work demonstrates that obstetric outcomes and management differ among these groups and the risk of pregnancy complications differs by the type of diabetes, being much higher in women with type 1 or type 2 diabetes than in women with GDM.^3^ We also examined the risk of both maternal and infant complications. Obstetric decision making is particularly complex, as benefits for the infant may increase the chance of harm to the mother, and vice versa.^32^ We also attempted to mimick the choices faced by care providers in their practice by examining outcomes following iatrogenic delivery or expectant management instead of examining outcomes for all women who delivered at a given week of gestation. Other work has shown that expectant management is not without harm, as women remain at risk of developing a hypertensive or infectious complication and, similarly, fetuses remain at risk of stillbirth.^32^

## 5 CONCLUSION

In conclusion, decisions about the timing of obstetric delivery are complex and must balance the risks and benefits to both the mother and fetus. This study demonstrates that there may be some benefit following delivery at 38, 39, or 40 weeks of gestation in women with diabetes. However, the fetus of a woman with poor glycemic control may need to be delivered earlier and the impact of the indication for earlier iatrogenic delivery may be the cause of the poorer outcome rather than the timing of the delivery. Ultimately, these decisions are best individualized and made in consultation with the patient and her care provider.

## 6 Funding 

This work was funded by an operating grant from the Canadian Institutes of Health Research (MOP‐142368). Amy Metcalfe is supported by a New Investigator Award and K. S. Joseph is supported by an Investigator award from the BC Children’s Hospital Research Institute. Parts of this material are based on data and information provided by the Canadian Institute for Health Information. Parts of this material are based on data and information provided by the Canadian Institute for Health Information. However, the analyses, conclusions, opinions and statements expressed herein are those of the author and not those of the Canadian Institute for Health Information.

## CONFLICT OF INTEREST

The authors have stated explicitly that there are no conflicts of interest in connection with this article.
