# Supplementary figures and images for: Timing of delivery in women with diabetes: A population‐based study
Source: Acta Obstet Gynecol Scand. 2019 Dec 8;99(3):341–9. doi: 10.1111/aogs.13761 (PMC7065101; doi:10.1111/aogs.13761)

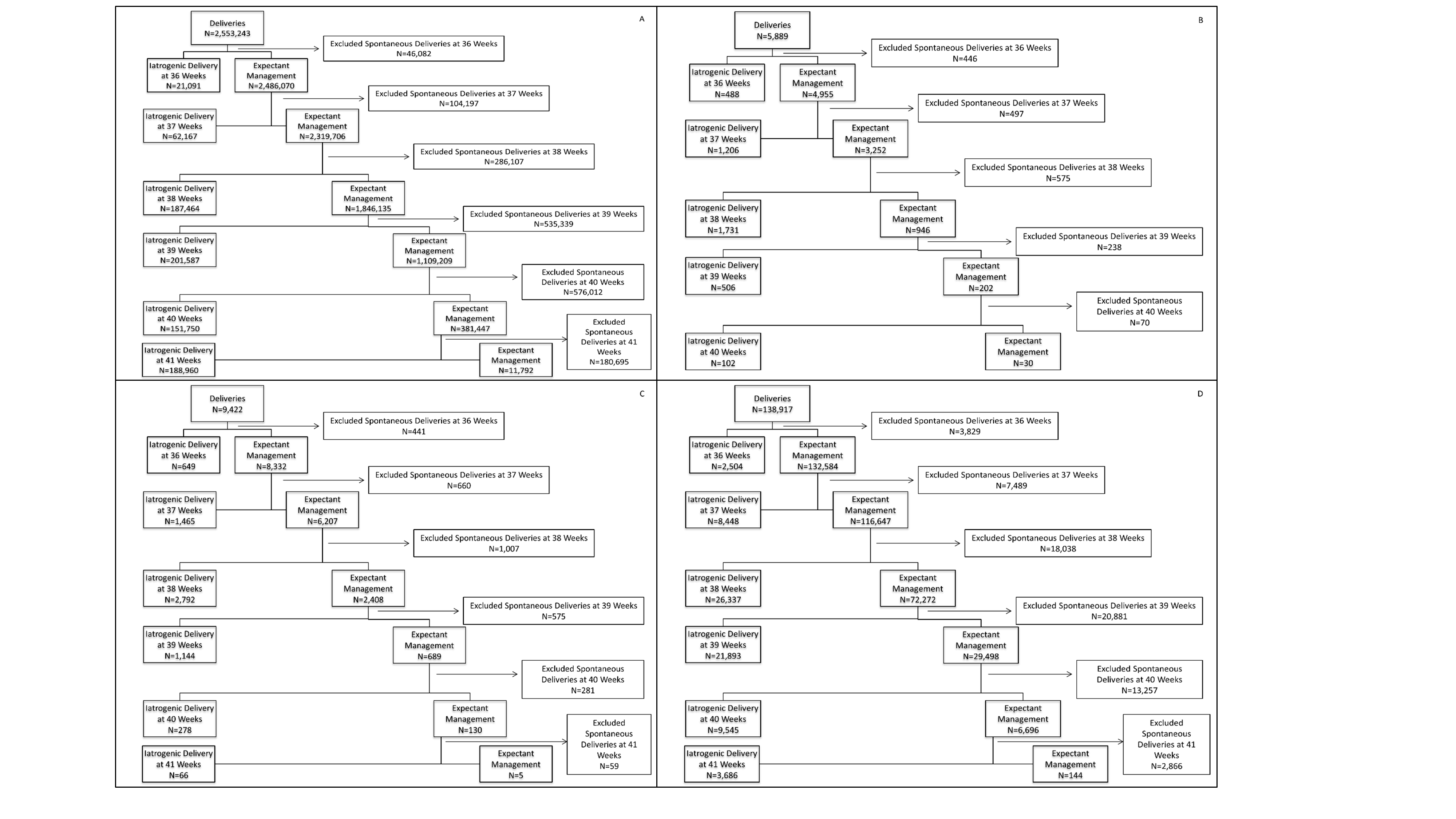

Supplement: Supplementary file 1 [file AOGS-99-341-s001.tif]
