# Supplementary material for: Timing of delivery in women with diabetes: A population‐based study
Source: Acta Obstet Gynecol Scand. 2019 Dec 8;99(3):341–9. doi: 10.1111/aogs.13761 (PMC7065101; doi:10.1111/aogs.13761)
Supplement: Supplementary file 2 [file AOGS-99-341-s002.docx]

Table S2. International Classification of Disease Version 10 Canadian Modification (ICD-10-CA) and Canadian Classification Intervention (CCI) Codes Used to Identify Study Population and Outcomes

| Condition | ICD-10-CA | CCI |
| --- | --- | --- |
| Obstetric delivery  Singleton live birth  Singleton stillbirth  Singleton born in hospital | Z37.0  Z37.1  Z38.0 |  |
| Congenital anomaly | Q00-Q99 |  |
| Labour induction |  | 5.AC.30.^^ |
| Cesarean section |  | 5.MD.60.^^ |
| Obstetric labour | O32.101, O33.xx1, O42.01, O42.02, O420.9, O42.11, O42.12, O42.19, O60.1, O61.x, O62.x, O63.x, O64.x, O65.x, O66.x, O68.x, O69.x, O71.10, O71.11, O71.18, O75.2, O75.3 |  |
| Maternal morbidity or mortality  Maternal death prior to discharge  Obstetric embolism  Obstetric shock  Post-partum hemorrhage with  hysterectomy or other  procedures to control bleeding  Sepsis  Thromboembolism    Uterine rupture | O95, O96, O97  O88  O75.1, R57, T80.5, T88.6  O72.0-O72.3  O75.3, O85  G08, I26, I80.1, I80.2, I80.3, I80.8, 180.9, I82, K55.0, K55.9, K75.1, N28.0, O07.2, O07.7, O08.2, O22.3, O22.8, O22.9, O87.1, O87.9, O88.2  O71.0, O71.1 | 5MD60KE, 5MD60RC, 5MD60CB, 5MD60RD, 1RM87LAGX, 1RM89LA, 5PC91LA, 1KT51, or 1RM13 without 1PL74, 1RS80, 1RS74 |
| Severe neonatal morbidity or mortality  Birth asphyxia  Fetal asphyxia  Intraventricular hemorrhage  (grades 3 or 4)  Neonatal convulsions  Other disturbances of cerebral  status of newborn  Respiratory distress syndrome  Birth injury to central nervous  system  Birth injury to peripheral  nervous system  Birth injury to skeleton  Fetal fracture of humerus or  clavicle to facilitate delivery  Shoulder dystocia  Stillbirth | P21  P20  P52.2  P90  P91  P22  P10, P11  P14  P13  O66.0  O36.4, P95, Z37.1 | 5.MD.45.QB, 5.MD.45.QC |

Table S3. Characteristics of deliveries to women delivering singleton non-anomalous infants ≥36 weeks of gestation in Canadian hospitals (excluding Quebec) between 2004-2014. Analysis restricted to first births during the study period.

| **Characteristic** | **Women Without Diabetes**  **n=1,755,265** | **Women with Type 1 Diabetes**  **n=3,982** | **Women with Type 2 Diabetes**  **n=6,059** | **Women with Gestational Diabetes**  **n=96,464** |
| --- | --- | --- | --- | --- |
| Maternal age (years), mean (sd) | 29.1 (5.7) | 29.5 (5.4) | 32.8 (5.6) | 32.0 (5.4) |
| Gestational age at delivery (weeks), mean (sd) | 39.3 (1.2) | 37.7 (1.0) | 38.0 (1.1) | 38.7 (1.1) |
| Iatrogenic delivery*,  % (95% CI) | 32.1 (32.0-32.2) | 74.0 (72.6-75.3) | 70.6 (69.5-71.8) | 52.3 (52.0-52.7) |
| Mode of delivery,  % (95% CI)  Spontaneous vaginal  Operative vaginal  Cesarean section | 60.9 (60.8-60.9)  12.7 (12.6-12.7)  26.5 (26.4-26.5) | 28.6 (27.2-30.0)  11.5 (10.6-12.6)  59.8 (58.3-61.3) | 38.6 (37.4-39.9)  8.8 (8.1-9.6)  52.5 (51.3-53.8) | 49.9 (49.6-50.2)  11.7 (11.5-11.9)  38.4 (38.1-38.7) |
| Hypertensive disorders of pregnancy, % (95% CI) | 5.4 (5.3-5.4) | 19.5 (18.3-20.8) | 16.9 (15.9-17.8) | 10.0 (9.8-10.2) |
| Parity, % (95% CI)  Nulliparous  Multiparous, no history of  prior cesarean section  Multiparous, prior cesarean  section | 55.3 (55.2-55.3)  34.6 (34.6-34.7)  10.1 (10.0-10.1) | 57.4 (55.6-59.1)  21.2 (19.8-22.7)  21.4 (20.0-22.9) | 39.6 (38.3-41.0)  38.0 (36.6-39.3)  22.4 (21.3-23.6) | 46.7 (46.3-47.0)  35.4 (35.0-35.7)  18.0 (17.7-18.2) |
| Diabetes complications severity index score, mean (sd) | --- | 3.6 (0.2) | 1.6 (0.2) | --- |
| Male infant sex, % (95% CI) | 50.6 (50.5-50.7) | 50.3 (48.7-51.9) | 50.0 (48.7-51.2) | 51.3 (51.0-51.6) |
| Infant birthweight (grams), mean (sd) | 3446.6 (475.8) | 3690.4 (602.2) | 3528.7 (614.4) | 3436.9 (517.9) |
| Large for gestational age infant >97^th^ percentile,  %, 95% CI | 8.6 (8.6-8.7) | 42.2 (40.6-43.7) | 27.9 (26.7-29.0) | 14.2 (14.0-14.4) |
| Severe maternal morbidity/mortality, % (95% CI) | 0.4 (0.4-0.4) | 0.6 (0.4-0.9) | 0.8 (0.6-1.1) | 0.5 (0.5-0.6) |
| Severe neonatal morbidity/mortality, % (95% CI) | 7.8 (7.7-7.8) | 19.9 (18.7-21.2) | 15.2 (14.4-16.2) | 9.7 (9.5-9.9) |

*defined as a delivery following induction of labour or pre-labour Cesarean delivery

Chi square tests were used to assess for differences between groups for categorical variables, while ANOVA and t-tests were used to assess for differences between groups for continuous variables.

Table S4. Risks of maternal morbidity/mortality by week of gestation for women with and without diabetes.

| **Type of Diabetes** | **Gestational Age** | **Predicted Adjusted Risk (95% CI) per 100 Deliveries Following Iatrogenic Delivery** | **Predicted Adjusted Risk (95% CI) per 100 Ongoing Pregnancies Following Expectant Management** | **Adjusted Risk Difference per 100 pregnancies (95% CI)** | **Adjusted Risk Ratio**  **(95% CI)** ^ʈ^ |
| --- | --- | --- | --- | --- | --- |
| No diabetes | 36 | 0.53 (0.46, 0.61) | 0.37 (0.36, 0.38) | **0.16 (0.09, 0.24)** | **1.44 (1.25, 1.67)** |
|  | 37 | 0.56 (0.51, 0.61) | 0.36 (0.35, 0.37) | **0.20 (0.15, 0.25)** | **1.55 (1.42, 1.70)** |
|  | 38 | 0.47 (0.44, 0.49) | 0.34 (0.33, 0.35) | **0.12 (0.09, 0.15)** | **1.36 (1.27, 1.46)** |
|  | 39 | 0.39 (0.36, 0.42) | 0.34 (0.30, 0.36) | **0.05 (0.02, 0.08)** | **1.14 (1.05, 1.23)** |
|  | 40 | 0.38 (0.35, 0.41) | 0.40 (0.38, 0.42) | -0.02 (-0.05, 0.02) | 0.96 (0.87, 1.06) |
|  | 41 | 0.40 (0.37, 0.43) | 0.62 (0.47, 0.76) | **-0.22 (-0.37, -0.07)** | **0.64 (0.51, 0.82)** |
| Type 1 Diabetes | 36 | 1.04 (0.11, 1.96) | 0.52 (0.32, 0.73) | 0.51 (-0.44, 1.46) | 1.98 (0.74, 5.27) |
|  | 37 | 0.33 (0.00, 0.66) | 0.52 (0.27, 0.77) | -0.19 (-0.60, 0.22) | 0.63 (0.21, 1.91) |
|  | 38 | 0.46 (0.14, 0.77) | 0.65 (0.13, 1.17) | -0.19 (-0.81, 0.42) | 0.70 (0.24, 2.05) |
|  | 39 | 0.99 (0.12, 1.86) | 0.49 (-0.48, 1.46) | 0.50 (-0.81, 1.81) | 2.01 (0.23, 17.49) |
|  | 40 | ---- | ---- | ---- | ---- |
|  | 41 | ---- | ---- | ---- | ---- |
| Type 2 Diabetes | 36 | 0.46 (0.00, 0.92) | 0.76 (0.57, 0.95) | -0.30 (-0.80, 0.19) | 0.60 (0.22, 1.68) |
|  | 37 | 0.60 (0.24, 0.97) | 0.77 (0.54, 0.99) | -0.16 (-0.60, 0.27) | 0.79 (0.40, 1.55) |
|  | 38 | 0.67 (0.38, 0.96) | 0.77 (0.40, 1.13) | -0.10 (-0.57, 0.37) | 0.87 (0.45, 1.67) |
|  | 39 | 0.58 (0.15, 1.01) | 1.27 (0.39, 2.16) | -0.69 (-1.68, 0.30) | 0.46 (0.16, 1.27) |
|  | 40 | 1.82 (0.06, 3.58) | 1.23 (-1.15, 3.61) | 0.59 (-2.40, 3.58) | 1.48 (0.17, 13.18) |
|  | 41 | ---- | ---- | ---- | ---- |
| Gestational Diabetes | 36 | 0.41 (0.20, 0.62) | 0.51 (0.47, 0.55) | -0.10 (-0.31, 0.12) | 0.81 (0.48, 1.36) |
|  | 37 | 0.60 (0.45, 0.74) | 0.49 (0.45, 0.53) | 0.10 (-0.05, 0.26) | 1.21 (0.93, 1.57) |
|  | 38 | 0.45 (0.37, 0.53) | 0.50 (0.45, 0.56) | -0.06 (-0.15, 0.04) | 0.89 (0.72, 1.09) |
|  | 39 | 0.47 (0.38, 0.56) | 0.56 (0.47, 0.65) | -0.09 (-0.22, 0.04) | 0.84 (0.65, 1.08) |
|  | 40 | 0.50 (0.36, 0.64) | 0.73 (0.52, 0.94) | -0.22 (-0.48, 0.03) | 0.69 (0.46, 1.04) |
|  | 41 | 0.62 (0.37, 0.88) | 0.70 (-0.67, 2.07) | -0.08 (-1.47, 1.32) | 0.89 (0.12, 6.54) |

^ʈ^The reference category is expectant management.

**Bold** text indicates statistically significant results.

Maternal death or severe maternal morbidity was defined as the occurrence of one or more of the following conditions/procedures in the immediate post-partum period: maternal death prior to discharge, obstetric embolism, obstetric shock, post-partum hemorrhage with hysterectomy or other procedures to control bleeding, sepsis, uterine rupture, or venous thromboembolism.

At each gestational age, the observed rate of each outcome was calculated per 100 deliveries following iatrogenic delivery and per 100 ongoing pregnancies following expectant management. Logistic regression models were derived to calculate adjusted risk ratios, risk differences, and absolute predicted risks for each outcome at each week of gestation. Models were adjusted for year, parity, and the obstetric comorbidity score.

Table S5. Risks of maternal morbidity/mortality by week of gestation for women with and without diabetes. Analysis restricted to first births during the study period.

| **Type of Diabetes** | **Gestational Age** | **Predicted Adjusted Risk (95% CI) per 100 Deliveries Following Iatrogenic Delivery** | **Predicted Adjusted Risk (95% CI) per 100 Ongoing Pregnancies Following Expectant Management** | **Adjusted Risk Difference per 100 pregnancies (95% CI)** | **Adjusted Risk Ratio**  **(95% CI)** ^ʈ^ |
| --- | --- | --- | --- | --- | --- |
| No diabetes | 36 | 0.53 (0.44, 0.62) | 0.39 (0.38, 0.40) | **0.14 (0.05, 0.23)** | **1.36 (1.15, 1.61)** |
|  | 37 | 0.57 (0.51, 0.63) | 0.38 (0.37, 0.39) | **0.19 (0.13, 0.25)** | **1.51 (1.35, 1.68)** |
|  | 38 | 0.52 (0.49, 0.56) | 0.36 (0.35, 0.37) | **0.17 (0.13, 0.21)** | **1.47 (1.35, 1.59)** |
|  | 39 | 0.43 (0.40, 0.47) | 0.36 (0.35, 0.38) | **0.07 (0.03, 0.10)** | **1.18 (1.08, 1.30)** |
|  | 40 | 0.40 (0.36, 0.44) | 0.41 (0.39, 0.44) | -0.02 (-0.06, 0.03) | 0.96 (0.86, 1.07) |
|  | 41 | 0.43 (0.39, 0.46) | 0.56 (0.41, 0.72) | 0.14 (-0.30, 0.02) | 0.76 (0.57, 1.01) |
| Type 1 Diabetes | 36 | 1.07 (0.00, 2.15) | 0.58 (0.33, 0.83) | 0.49 (-0.62, 1.60) | 1.85 (0.62, 5.52) |
|  | 37 | 0.45 (0.01, 0.90) | 0.60 (0.28, 0.91) | -0.14 (-0.69, 0.40) | 0.76 (0.25, 2.33) |
|  | 38 | 0.55 (0.14, 0.96) | 0.75 (0.08, 1.42) | -0.21 (-0.10, 0.59) | 0.73 (0.23, 2.35) |
|  | 39 | 1.02 (0.02, 2.03) | 0.71 (-0.69, 2.11) | 0.32 (-1.42, 2.06) | 1.45 (0.16, 13.42) |
|  | 40 | ---- | ---- | ---- | ---- |
|  | 41 | ---- | ---- | ---- | ---- |
| Type 2 Diabetes | 36 | 0.56 (0.00, 1.19) | 0.83 (0.59, 1.07) | -0.28 (-0.96, 0.41) | 0.67 (0.20, 2.18) |
|  | 37 | 0.66 (0.19, 1.13) | 0.85 (0.56, 1.13) | -0.19 (-0.74, 0.37) | 0.78 (0.35, 1.72) |
|  | 38 | 0.74 (0.37, 1.11) | 0.71 (0.29, 1.13) | 0.03 (-0.54, 0.60) | 1.04 (0.47, 2.28) |
|  | 39 | 0.60 (0.01, 1.18) | 1.48 (0.17, 2.80) | -0.89 (-2.33, 0.56) | 0.40 (0.11, 1.53) |
|  | 40 | 1.61 (-0.14, 3.37) | 2.20 (-1.78, 6.18) | -0.59 (-0.05, 3.83) | 0.73 (0.09, 6.29) |
|  | 41 | ---- | ---- | ---- | ---- |
| Gestational Diabetes | 36 | 0.37 (0.14, 0.61) | 0.53 (0.48, 0.57) | -0.15 (-0.39, 0.08) | 0.71 (0.38, 1.33) |
|  | 37 | 0.71 (0.52, 0.91) | 0.51 (0.46, 0.56) | 0.21 (0.00, 0.41) | **1.41 (1.05, 1.88)** |
|  | 38 | 0.48 (0.38, 0.58) | 0.51 (0.45, 0.57) | -0.03 (-0.15, 0.09) | 0.94 (0.74, 1.20) |
|  | 39 | 0.51 (0.39, 0.62) | 0.56 (0.46, 0.67) | -0.06 (-0.21, 0.10) | 0.90 (0.67, 1.20) |
|  | 40 | 0.49 (0.33, 0.65) | 0.77 (0.53, 1.01) | -0.28 (-0.57, 0.01) | 0.63 (0.40, 1.01) |
|  | 41 | 0.69 (0.39, 0.99) | 0.88 (-0.84, 2.60) | -0.19 (-1.93, 1.55) | 0.78 (0.11, 5.79) |

^ʈ^The reference category is expectant management.

**Bold** text indicates statistically significant results.

Maternal death or severe maternal morbidity was defined as the occurrence of one or more of the following conditions/procedures in the immediate post-partum period: maternal death prior to discharge, obstetric embolism, obstetric shock, post-partum hemorrhage with hysterectomy or other procedures to control bleeding, sepsis, uterine rupture, or venous thromboembolism.

At each gestational age, the observed rate of each outcome was calculated per 100 deliveries following iatrogenic delivery and per 100 ongoing pregnancies following expectant management. Logistic regression models were derived to calculate adjusted risk ratios, risk differences, and absolute predicted risks for each outcome at each week of gestation. Models were adjusted for year, parity, and the obstetric comorbidity score.

Table S6. Risks of neonatal morbidity and mortality by week of gestation for infants born to women with and without diabetes.

| **Type of Diabetes** | **Gestational Age** | **Predicted Adjusted Risk (95% CI) per 100 Deliveries Following Iatrogenic Delivery** | **Predicted Adjusted Risk (95% CI) per 100 Ongoing Pregnancies Following Expectant Management** | **Adjusted Risk Difference per 100 pregnancies (95% CI)** | **Adjusted Risk Ratio**  **(95% CI)** ^ʈ^ |
| --- | --- | --- | --- | --- | --- |
| No diabetes | 36 | 15.72 (15.22, 16.22) | 7.60 (7.56, 7.63) | **8.13 (7.62, 8.63)** | **2.07 (2.00, 2.14)** |
|  | 37 | 10.13 (9.89, 10.37) | 7.54 (7.50, 7.57) | **2.59 (2.35, 2.84)** | **1.34 (1.31, 1.38)** |
|  | 38 | 6.91 (6.79, 7.03) | 7.80 (7.76, 7.84) | **-0.89 (-1.02, -0.76)** | **0.89 (0.87, 0.90)** |
|  | 39 | 6.11 (6.00, 6.22) | 8.63 (8.57, 8.68) | **-2.51 (-2.64, -2.39)** | **0.71 (0.70, 0.72)** |
|  | 40 | 8.43 (8.29, 8.57) | 9.51 (9.41, 9.60) | **-1.08 (-1.25, -0.90)** | **0.89 (0.87, 0.90)** |
|  | 41 | 9.64 (9.50, 9.77) | 10.05 (9.49, 10.60) | -0.41 (-0.98, 0.16) | 0.96 (0.91, 1.02) |
| Type 1 Diabetes | 36 | 28.70 (24.48, 32.92) | 18.87 (17.77, 19.98) | **9.83 (5.45, 14.20)** | **1.52 (1.30, 1.78)** |
|  | 37 | 21.55 (19.16, 23.94) | 17.10 (15.79, 18.42) | **4.45 (1.71, 7.18)** | **1.26 (1.10, 1.44)** |
|  | 38 | 17.87 (16.03, 19.71) | 15.17 (12.84, 17.50) | 2.70 (-0.29, 5.69) | 1.18 (0.98, 1.42) |
|  | 39 | 14.96 (11.79, 18.13) | 12.80 (8.14, 17.47) | 2.16 (-3.54, 7.86) | 1.17 (0.76, 1.79) |
|  | 40 | 12.77 (6.37, 19.17) | 10.27 (-0.67, 21.22) | 2.50 (-10.23, 15.23) | 1.24 (0.38, 4.05) |
|  | 41 | ---- | ---- | ---- | ---- |
| Type 2 Diabetes | 36 | 21.93 (18.58, 25.27) | 14.14 (13.38, 14.90) | **7.79 (4.36, 11.22)** | **1.55 (1.32, 1.82)** |
|  | 37 | 15.40 (13.48, 17.33) | 13.48 (12.62, 14.34) | 1.93 (-0.19, 4.04) | 1.14 (0.99, 1.32) |
|  | 38 | 14.07 (12.74, 15.39) | 12.90 (11.54, 14.26) | 1.17 (-0.74, 3.08) | 1.09 (0.95, 1.26) |
|  | 39 | 12.71 (10.73, 14.69) | 14.58 (11.85, 17.32) | -1.88 (-5.29, 1.54) | 0.87 (0.68, 1.12) |
|  | 40 | 14.55 (10.35, 18.76) | 14.17 (8.09, 20.25) | 0.39 (-7.05, 7.83) | 1.03 (0.61, 1.73) |
|  | 41 | 11.38 (3.57, 19.18) | 18.22 (-13.98, 50.41) | -6.84 (-40.08, 26.39) | 0.62 (0.09, 4.20) |
| Gestational Diabetes | 36 | 17.20 (15.67, 18.73) | 9.53 (9.37, 9.69) | **7.67 (6.13, 9.21)** | **1.80 (1.65, 1.98)** |
|  | 37 | 12.10 (11.38, 12.82) | 9.32 (9.15, 9.49) | **2.78 (2.04, 3.52)** | **1.30 (1.22, 1.38)** |
|  | 38 | 8.85 (8.49, 9.20) | 9.64 (9.42, 9.86) | **-0.79 (-1.22, -0.37)** | **0.92 (0.88, 0.96)** |
|  | 39 | 8.26 (7.88, 8.63) | 10.99 (10.62, 11.36) | **-2.73 (-3.27, -2.20)** | **0.75 (0.71, 0.80)** |
|  | 40 | 10.23 (9.61, 10.85) | 11.82 (11.03, 12.61) | **-1.59 (-2.60, -0.58)** | **0.87 (0.79, 0.95)** |
|  | 41 | 10.84 (9.83, 11.86) | 15.32 (9.44, 21.21) | -4.48 (-10.45, 1.49) | 0.71 (0.48, 1.05) |

^ʈ^The reference category is expectant management.

**Bold** text indicates statistically significant results.

Severe neonatal morbidity or mortality was defined as the occurrence of one or more of the following conditions/procedures during the birth hospitalization: birth asphyxia, fetal asphyxia, intraventricular hemorrhage (grade 3 or 4), neonatal convulsions, other disturbances of cerebral status of newborn, respiratory distress syndrome, birth injury to central nervous system, birth injury to peripheral nervous system, birth injury to skeleton, fetal fracture of humerus or clavicle to facilitate delivery, shoulder dystocia, stillbirth or neonatal death.

At each gestational age, the observed rate of each outcome was calculated per 100 deliveries following iatrogenic delivery and per 100 ongoing pregnancies following expectant management. Logistic regression models were derived to calculate adjusted risk ratios, risk differences, and absolute predicted risks for each outcome at each week of gestation. Models were adjusted for year, parity, obstetric comorbidity score and infant sex.

Table S7. Risks of neonatal morbidity and mortality by week of gestation for infants born to women with and without diabetes. Analysis restricted to first births during the study period only.

| **Type of Diabetes** | **Gestational Age** | **Predicted Adjusted Risk (95% CI) per 100 Deliveries Following Iatrogenic Delivery** | **Predicted Adjusted Risk (95% CI) per 100 Ongoing Pregnancies Following Expectant Management** | **Adjusted Risk Difference per 100 pregnancies (95% CI)** | **Adjusted Risk Ratio**  **(95% CI)** ^ʈ^ |
| --- | --- | --- | --- | --- | --- |
| No diabetes | 36 | 15.47 (14.90, 16.05) | 7.80 (7.76, 7.84) | **7.67 (7.10, 8.25)** | **1.98 (1.91, 2.06)** |
|  | 37 | 10.14 (9.84, 10.43) | 7.74 (7.70, 7.78) | **2.40 (2.10, 2.69)** | **1.31 (1.27, 1.35)** |
|  | 38 | 7.12 (6.96, 7.27) | 7.99 (7.95, 8.04) | **-0.88 (-1.04, -0.71)** | **0.89 (0.87, 0.91)** |
|  | 39 | 6.55 (6.41, 6.70) | 8.72 (8.65, 8.78) | **-2.17 (-2.32, -2.01)** | **0.75 (0.73, 0.77)** |
|  | 40 | 8.52 (8.35, 8.69) | 9.54 (9.43, 9.65) | **-1.02 (-1.23, -0.82)** | **0.89 (0.87, 0.91)** |
|  | 41 | 9.62 (9.46, 9.77) | 9.88 (9.25, 10.51) | -0.26 (-0.91, 0.38) | 0.97 (0.91, 1.04) |
| Type 1 Diabetes | 36 | 28.17 (23.37, 32.97) | 19.07 (17.77, 20.37) | **9.10 (4.10, 14.09)** | **1.48 (1.23, 1.78)** |
|  | 37 | 21.75 (18.93, 24.58) | 17.51 (15.96, 19.07) | **4.24 (1.00, 7.48)** | **1.24 (1.06, 1.45)** |
|  | 38 | 18.73 (16.51, 20.95) | 14.43 (11.76, 17.10) | **4.30 (0.80, 7.81)** | **1.30 (1.04, 1.62)** |
|  | 39 | 13.64 (10.10, 17.18) | 13.96 (8.29, 19.63) | -0.32 (-7.09, 6.45) | 0.98 (0.60, 1.59) |
|  | 40 | 12.86 (5.65, 20.06) | 11.88 (-0.57, 24.33) | 0.98 (-13.51, 15.46) | 1.08 (0.33, 3.58) |
|  | 41 | ---- | ---- | ---- | ---- |
| Type 2 Diabetes | 36 | 23.18 (18.86, 27.51) | 14.01 (13.09, 14.93) | **9.17 (4.76, 13.59)** | **1.65 (1.36, 2.02)** |
|  | 37 | 15.54 (13.13, 17.96) | 13.24 (12.21, 14.28) | 2.30 (-0.33, 4.93) | 1.17 (0.99, 1.40) |
|  | 38 | 14.38 (12.74, 16.03) | 12.51 (10.92, 14.11) | 1.87 (-0.44, 4.18) | 1.15 (0.97, 1.37) |
|  | 39 | 11.92 (9.61, 14.24) | 14.93 (11.73, 18.14) | -3.01 (-7.01, 0.99) | 0.80 (0.60, 1.07) |
|  | 40 | 14.68 (9.85, 19.51) | 17.24 (9.76, 24.72) | -2.56 (-11.52, 6.41) | 0.85 (0.49, 1.47) |
|  | 41 | 15.59 (5.50, 25.69) | 19.75 (-14.82, 54.32) | -4.16 (-40.39, 32.07) | 0.79 (0.12, 5.17) |
| Gestational Diabetes | 36 | 16.37 (14.61, 18.14) | 9.56 (9.37, 9.75) | **6.81 (5.04, 8.59)** | **1.71 (1.54, 1.91)** |
|  | 37 | 12.08 (11.20, 12.95) | 9.37 (9.17, 9.57) | **2.70 (1.81, 3.60)** | **1.29 (1.19, 1.39)** |
|  | 38 | 8.97 (8.53, 9.41) | 9.67 (9.41, 9.92) | **-0.70 (-1.21, -0.18)** | **0.93 (0.88, 0.98)** |
|  | 39 | 8.55 (8.08, 9.02) | 10.82 (10.40, 11.24) | **-2.27 (-2.91, -1.63)** | **0.79 (0.74, 0.85)** |
|  | 40 | 9.96 (9.26, 10.66) | 11.61 (10.73, 12.49) | **-1.65 (-2.78, -0.51)** | **0.86 (0.77, 0.95)** |
|  | 41 | 10.51 (9.38, 11.64) | 16.68 (9.82, 23.53) | -6.16 (-13.11, 0.78) | **0.63 (0.41, 0.96)** |

^ʈ^The reference category is expectant management.

**Bold** text indicates statistically significant results.

Severe neonatal morbidity or mortality was defined as the occurrence of one or more of the following conditions/procedures during the birth hospitalization: birth asphyxia, fetal asphyxia, intraventricular hemorrhage (grade 3 or 4), neonatal convulsions, other disturbances of cerebral status of newborn, respiratory distress syndrome, birth injury to central nervous system, birth injury to peripheral nervous system, birth injury to skeleton, fetal fracture of humerus or clavicle to facilitate delivery, shoulder dystocia, stillbirth or neonatal death.

At each gestational age, the observed rate of each outcome was calculated per 100 deliveries following iatrogenic delivery and per 100 ongoing pregnancies following expectant management. Logistic regression models were derived to calculate adjusted risk ratios, risk differences, and absolute predicted risks for each outcome at each week of gestation. Models were adjusted for year, parity, obstetric comorbidity score and infant sex.
